# Insights into toxic *Prymnesium parvum* blooms: the role of sugars and algal viruses

**DOI:** 10.1042/BST20170393

**Published:** 2018-03-14

**Authors:** Ben A. Wagstaff, Edward S. Hems, Martin Rejzek, Jennifer Pratscher, Elliot Brooks, Sakonwan Kuhaudomlarp, Ellis C. O'Neill, Matthew I. Donaldson, Steven Lane, John Currie, Andrew M. Hindes, Gill Malin, J. Colin Murrell, Robert A. Field

**Affiliations:** 1 Department of Biological Chemistry, John Innes Centre, Norwich Research Park, Norwich NR4 7UH, U.K.; 2 The Lyell Centre, Heriot-Watt University, Edinburgh EH14 4AS, U.K.; 3 Centre for Ocean and Atmospheric Sciences, School of Environmental Sciences, University of East Anglia, Norwich Research Park, Norwich NR4 7TJ, U.K.; 4 Department of Plant Sciences, University of Oxford, Oxford OX1 3RB, U.K.; 5 Fisheries, Biodiversity and Geomorphology Team, Environment Agency, Dragonfly House, Norwich NR3 1UB, U.K.; 6 Pike Anglers’ Club, 1 Kirby House Cottages, Norwich NR14 7DZ, U.K.; 7 Fishtrack Ltd, 2 South End Farm Cottages, Beccles NR34 8TG, U.K.

**Keywords:** algal toxins, algal virus, harmful algal blooms, Norfolk Broads, *Prymnesium parvum*

## Abstract

*Prymnesium parvum* is a toxin-producing microalga that causes harmful algal blooms globally, which often result in large-scale fish kills that have severe ecological and economic implications. Although many toxins have previously been isolated from *P. parvum*, ambiguity still surrounds the responsible ichthyotoxins in *P. parvum* blooms and the biotic and abiotic factors that promote bloom toxicity. A major fish kill attributed to *P. parvum* occurred in Spring 2015 on the Norfolk Broads, a low-lying set of channels and lakes (Broads) found on the East of England. Here, we discuss how water samples taken during this bloom have led to diverse scientific advances ranging from toxin analysis to discovery of a new lytic virus of *P. parvum*, *P. parvum* DNA virus (PpDNAV-BW1). Taking recent literature into account, we propose key roles for sialic acids in this type of viral infection. Finally, we discuss recent practical detection and management strategies for controlling these devastating blooms.

## Introduction

Harmful algal blooms (HABs) are rapid expansions of phytoplankton populations, which represent a major threat to the health of diverse coastal and freshwater aquatic ecosystems [1]. Commonly, these algal blooms are dominated by one or a few phytoplankton species, and damage to the surrounding ecosystem can occur via several different mechanisms. Eutrophication, which is probably the best known cause of HABs, leads to water hypoxia through the bacterial-mediated decomposition of dead algal blooms. However, mechanical gill damage and production of algal toxins represent two further mechanisms through which aquatic life can suffer [2]. The frequency of HABs appears to have increased in recent years, perhaps due to climate change [3]; as a consequence, there has been an increased focus from both scientists and regulatory authorities to combat the negative effects of HABs. While regulators have focused on practical mitigation or management strategies, scientists have sought to learn more about what promotes HABs and the toxin-producing species that cause them [4]. *Prymnesium parvum* is one such toxin-producing microalga that causes HABs globally, resulting in large-scale fish mortalities that have negative effects on ecosystems and the economy of the affected areas [5]. Research into *P. parvum* has been ongoing since blooms by this organism were first reported in the Netherlands by Liebert and Deerns in 1920 [6]. Since then, blooms of *P. parvum* have been reported worldwide, with examples of mass fish kills found in Scotland [7], Norway [8], Germany [9], Finland [10], China [11], and the U.S.A. [12], where it is of particular concern to the aquaculture industry [13]. Edvardsen and Paasche [14] have also commented on blooms of *P. parvum* in Israel, former USSR, Bulgaria, Spain, Denmark, Sweden, and Australia. However, our major interest in *P. parvum* was galvanized by the effects of repeated blooms of this microalga in our area in the Norfolk Broads in the East of England [15].

The Norfolk Broads are a low-lying area of interconnected rivers, channels, and lakes (Broads) that are a popular tourist destination for angling and boating activities. Originally excavated for peat and fuel prior to the 14th century, the Broads and their surrounding marshland now house a plethora of rare wildlife and represent Britain's largest protected wetland [16]. Up until the 1960s, much of the Broads were dominated by a healthy charophyte-based algal community [15]. Hickling Broad, in particular, then underwent a change to a phytoplankton-dominated community through the 1960s and 1970s. While charophytes have returned and are a factor in the Broads being a Designated Special Area of Conservation, Hickling and the surrounding Broads still suffer from almost annual occurrences of *P. parvum* blooms [17]. These toxic blooms frequently lead to mass fish mortalities that threaten the ecosystem of this national park and the estimated £550 million of annual revenue it generates through tourism [16].

Although significant effort has gone into researching the bloom dynamics of *P. parvum* [5], this new knowledge has not yet translated into feasible, practical solutions for bloom prevention or management. Here, we discuss recent advances in both the scientific understanding, and control and management, of *P. parvum* blooms on the Norfolk Broads. Facilitated by a major bloom of *P. parvum* in Spring 2015, scientists and governing bodies across Norfolk collaborated to tackle the issue head on.

## *P. parvum* and its toxins

**Figure 1.**
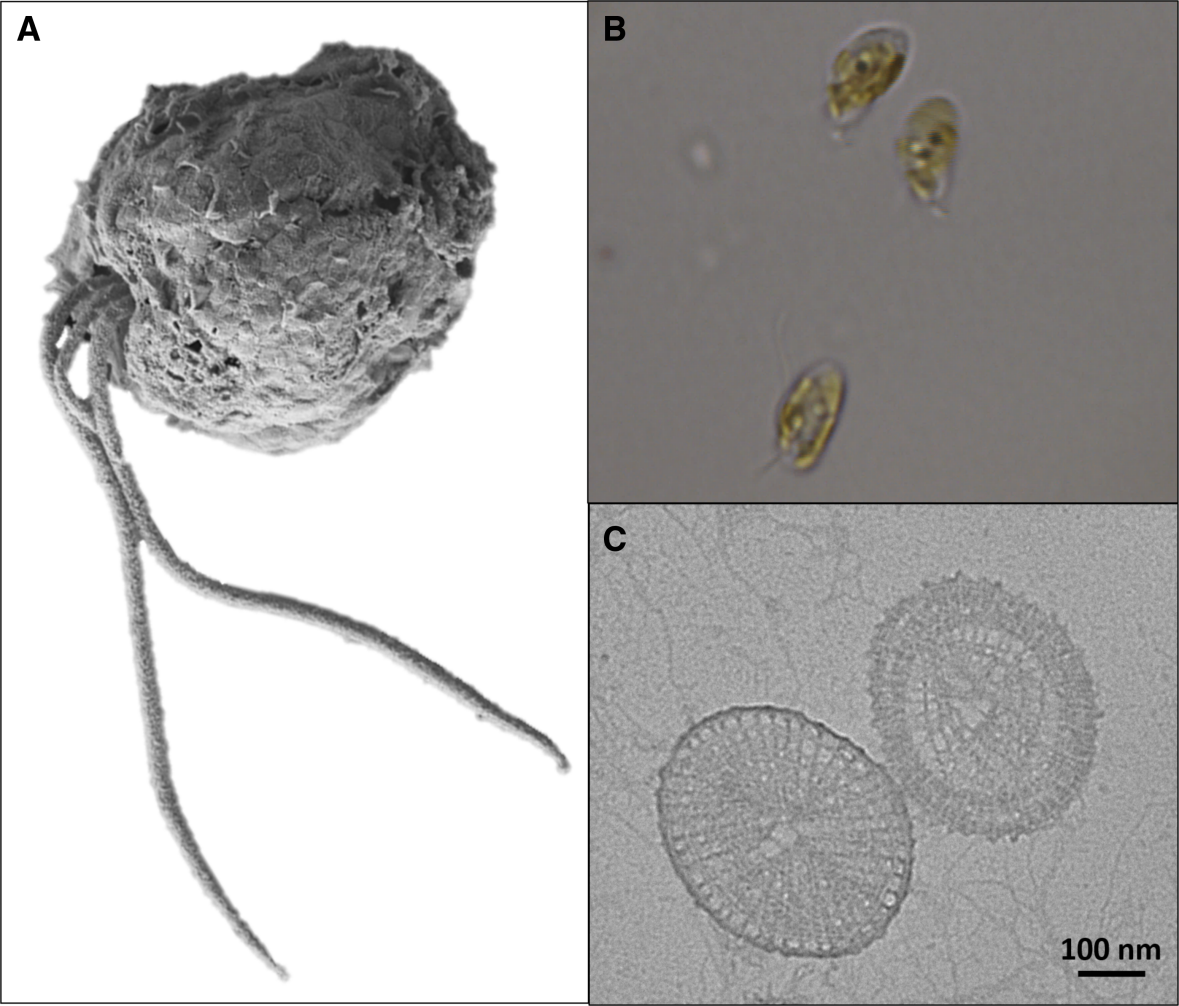
Fine morphology of *P. parvum*. (**A**) *P. parvum* (CCAP 946/6) cell observed by SEM (background digitally removed). Note the presence of two long flagella and the shorter central haptonema. (**B**) Three *P. parvum* cells observed by optical microscopy using a Leica DM6000 fitted with a 100× objective. Cells show the golden colour typically associated with blooms of the organism. (**C**) Scales of *P. parvum* observed by transmission electron microscopy (TEM). Scale bar represents 100 nm.

Commonly referred to as a golden alga due to the fucoxanthin pigments found in its chloroplasts [18], *P. parvum* is a unicellular microalga belonging to the Prymnesiaceae of the phylum Haptophyta [19]. Two long flagella permit movement and stir the boundary layer around the cells to aid nutrient uptake, while a shorter haptonema is used for attachment to prey in the phagocytic process, helping *P. parvum* perform as a successful mixotroph [20,21] (Figure 1). Like other members of the Prymnesiales, *P. parvum* has organic scales covering the outer cell membrane that are often used for phylogenetic analysis due to their unique appearance [22] (Figure 1C). Its success as a cosmopolitan organism is, in part, due to the euryhaline and eurythermal nature of the organism, tolerating salinities ranging from 3 PSU (just above freshwater) to 30 PSU (sea water) [23,24], and temperatures from 2 to 30°C [24,25].

**Figure 2.**
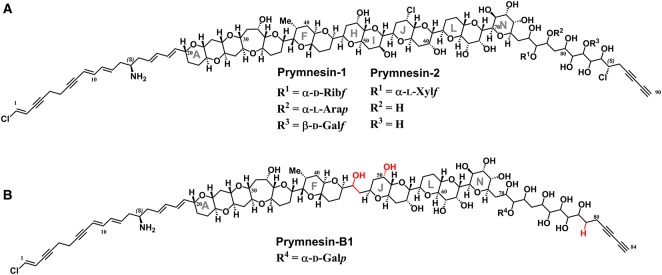
Structures of ladder-frame polyether prymnesins-1, -2, and -B1. (**A**) Structure of prymnesin-1 and -2 first reported by Igarashi et al. [35], incorporating amended structural information by Sasaki et al. [38]. (**B**) Structure of prymnesin-B1 (notice the lack of rings H and I) recently reported by Rasmussen et al. [34], with areas of the backbone highlighted red that differ from prymnesin-1 and -2.

Toxins reported to be produced by *P. parvum* are diverse and include lipopolysaccharide-like compounds [26], proteolipid [27], galactoglycerolipids [28], fatty acid amides [29,30], fatty acids [31], and the ladder-frame polyether prymnesins [32–34]. First isolated and characterized in two forms (prymnesin-1 and -2) in 1995 by Igarashi et al. [32,33,35], the diversity of these potent nanomolar ichthyotoxins has recently expanded to include prymnesin-B1 and others with slight variations in structure to the originally isolated compounds [34] (Figure 2). However, because of the minute amounts of these toxins produced by the organism, detection of these compounds represents a major challenge [34,36]. The current ambiguity on the responsible toxins in Prymnesium-associated fish mortality has meant that toxins have been proposed to be both intra- and extracellular. However, Remmel and Hambright [37] suggested that toxins are intracellular, and only released through contact with prey or physical stress.

## Open questions about *P. parvum* bloom toxicity

- First, although it is generally accepted that the ladder-frame polyether prymnesins are responsible for fish mortality, the lack of detection of these toxins in environmental water samples has put their significance into question. As a result, it is currently unclear whether these toxins are the primary ichthyotoxins in *P. parvum* blooms.
- Second, although significant research has focused on how a range of abiotic factors affect the production and toxicity of *P. parvum* blooms, there have been few clear links in natural waterways that attribute a specific abiotic factor (nutrients, temperature and pH) to increased bloom toxicity (reviewed by Manning and La Claire [5]). Therefore, does an unknown environmental factor trigger bloom toxicity?
- Third, because of the ambiguity in the responsible toxins, it is currently unknown whether *P. parvum* toxins are intra- or extracellular toxins. If they are intracellular, how are they released into the waterways?

## Detection of *P. parvum* toxins

Ladder-frame polyether prymnesins-1 and -2 were first isolated and had their structures elucidated in the 1990s by Igarashi et al. [32,33,35] and later Sasaki et al. [38]. Despite this, there was at least a 10-year gap before other researchers reported the detection of these toxins in laboratory cultures of *P. parvum*. This gap led to much speculation about the significance of the ladder-frame polyether prymnesins, with researchers looking elsewhere for responsible ichthyotoxins [30]. In 2013, Manning et al. reported a detailed extraction and a LC–MS protocol for the detection of prymnesin-1 and -2 from laboratory cultures [36], but detection of the toxins in environmental water samples still had not been reported, despite recurring worldwide blooms of *P. parvum* in this period. Most recently, in 2016, Rasmussen et al. [34] reported a previously unknown structural diversity of the prymnesins isolated from different strains of *P. parvum*. They proposed that this structural diversity had meant that researchers were looking for the wrong metabolic fingerprints when analyzing water samples.

A toxic bloom of *P. parvum* in Hickling Broad in Spring 2015 allowed us to follow the extraction and LC–MS methods outlined by Manning and La Claire [36] for the detection of these toxins. However, neither prymnesin-1 nor -2 could be observed in water samples collected during this bloom event (unpublished observations). However, a more thorough analysis of our LC–MS data, combined with the details of the new prymnesin toxins reported by Rasmussen et al. [34], has led to our detecting the ladder-frame polyether prymnesins in Broads water samples for the first time (manuscript in preparation). These findings suggest that the previous inability to detect the ladder-frame polyether prymnesin toxins was not because of the low amounts in natural waters, but rather due to the fact that researchers were previously looking for a narrow (and often incorrect) window of metabolic fingerprints in many instances, as previously proposed by Rasmussen et al. [34].

## Discovery of a lytic virus of *P. parvum*

**Figure 3.**
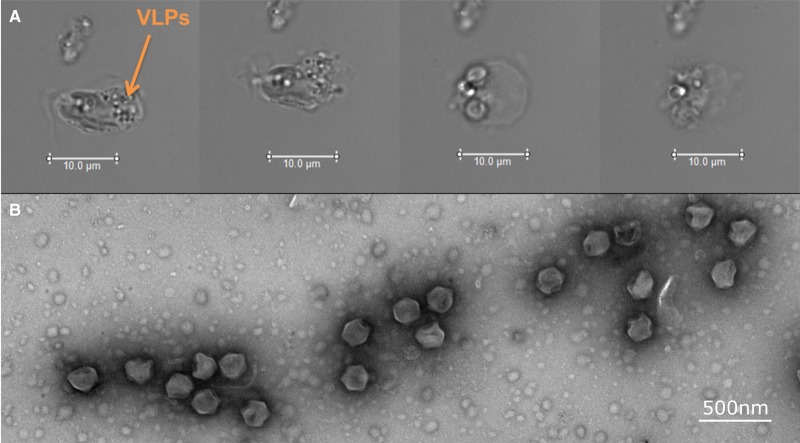
Viral infection of natural *P. parvum* and discovery of PpDNAV-BW1. (**A**) A natural *P. parvum* cell taken from water samples from Hickling Broad during a toxic bloom in Spring 2015. Light microscopy was used to capture images over a 4-h period and show (from left to right) a non-motile cell filled with putative virus-like particles (VLPs) undergoing membrane blebbing before bursting and releasing intracellular contents. Scale bars represent 10 µm. (**B**) TEM images of *P. parvum* DNA virus (PpDNAV-BW1). Scale bar represents 500 nm.

The last two decades have seen an increase in the study of algal viruses and the role that they play in the regulation of algal bloom dynamics [39]. Typically 100–220 nm in diameter, and with genomes up to 560 kb [40], dsDNA algal viruses such as the Phycodnaviridae have also been shown to contribute significantly to global biogeochemical cycles [41,42]. Much less studied, however, is the role that viruses play in the regulation of algal blooms by toxin-producing species. During the toxic *P. parvum* bloom on Hickling Broad in 2015, optical microscopy of the native population of *P. parvum* suggested that it was infected by a virus (Figure 3A). This subsequently led to the isolation of a new lytic virus from Hickling Broad that infects *P. parvum*, *P. parvum* DNA virus (PpDNAV-BW1) [43] (Figure 3B). The host range of this virus was screened against 15 strains of Prymnesium and found to infect 5 out of the 15. A narrow host range is typical for algal viruses but not always the case as shown and discussed by Johannessen et al. [44], and this specificity may suggest intricate molecular mechanisms behind viral infection of these algae. Electron microscopy showed that the average capsid diameter size was 221 nm, and an initial genome assembly (ongoing investigation) suggests that it has a genome size of ∼500 kb and belongs to the algal Megaviridae family.

Previously, mechanical breakdown of cells by biotic factors, such as grazers and viruses, have been proposed to be a potential exit route of intracellular algal toxins [37]. Although viruses infecting toxin-producing microalgae have been discovered previously [45–47], *P. parvum* and its associated virus represent the first system where the toxins produced by the host alga are fully characterized and detectable in laboratory cultures. This *P. parvum *: PpDNAV-BW1 system may therefore provide a platform to answer fundamental questions surrounding the effect of viral infection on toxin production and release in microalgae.

## Insights into the molecular basis for viral recognition and infection of *P. parvum* by PpDNAV-BW1

**Figure 4.**
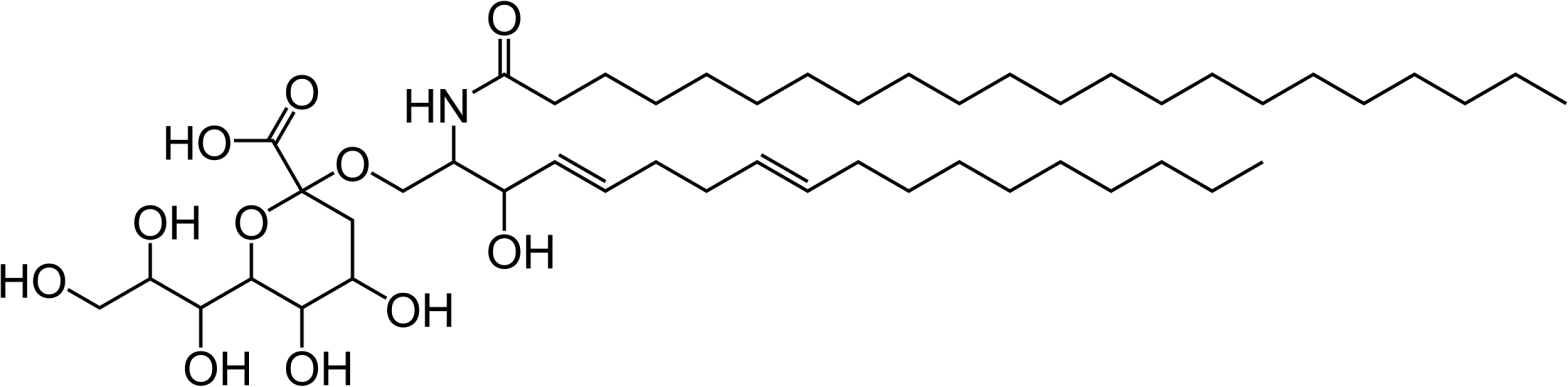
Tentative structure of a sialic acid-containing sphingolipid from the haptophyte *E. huxleyi*. A novel sphingolipid isolated from *E. huxleyi* with a polar head group containing the deaminated sialic acid, 2-keto-3-deoxy-d-glycero-d-galacto-nononic acid (KDN). Structure redrawn from Fulton et al. [49].

Recent work has highlighted sialic acids as mediators of viral infection of the haptophyte *Emiliania huxleyi* [48,49] (Figure 4). Sialic acids are acidic, nine-carbon carbohydrates that are found in several kingdoms, including on the surface of all vertebrate cells [50]. Most often, sialic acids occupy the terminal residue of a glycan on a cell surface, meaning that they are exposed to a range of host–pathogen interactions [51]. Sialic acid involvement in viral infections of other organisms is not unknown; probably, the best studied example is the binding of the human or avian influenza viruses to sialic acids on epithelial cells of its host [52]. We have previously exploited this highly specific molecular interaction to develop novel diagnostics that distinguish between human and avian influenza viruses [53].

The production of sialic acids by algae was undocumented prior to the recent work on *E. huxleyi* by Rose et al. [48]. We therefore sought to investigate the presence or otherwise of sialic acids in *P. parvum* and algae more broadly. One way of analyzing sialic acids in a host is using sialic acid-binding proteins (SIGLECs) [54]. Although these assays are relatively cheap and simple to perform, they are often specific for a given type and sugar-linkage of sialic acid. More detailed glycan analysis is often performed using a range of mass spectrometry techniques (reviewed by Mulloy et al. [55]), although these are more labour-intensive and -expensive. Profiling of the nucleotide-activated sugars inside the cell can give detailed insights into the sugars an organism is capable of producing [56], although this does not confirm the final destination of the sugar, which can range from natural products through to glycans, glycoproteins, polysaccharides, or glycolipids. Finally, analysis of carbohydrate active enzyme (CAZyme) sequence information can often allude to the types of sugars produced, and in many cases, the glycan structures produced [57]. We have previously applied all these techniques to look for sialic acids and other sugars in the green alga *Euglena gracilis* [58] and are now applying these techniques to investigate sialic acid production in *P. parvum*, with a view to deciphering its importance in viral infection by PpDNAV-BW1. Preliminary results suggest that *P. parvum* produces a sialic acid, and that sialic acid production is more widespread among algae than previously thought (ongoing investigation).

## Future management of *P. parvum* blooms

The occurrence of HABs poses a severe threat to ecosystems, economies, and in some cases human and animal health. Therefore, there is a great need to develop practical detection, mitigation, and management strategies for HABs.

For the detection and monitoring of algal populations, governing bodies typically rely on optical microscopy. In such diverse phytoplankton communities, optical microscopy can be challenging for even the most skilled phycologist. Furthermore, these methods are often time-consuming and less accurate than alternative molecular methods. For the detection of algal toxins, animal bioassays are frequently employed, although these are associated with both technical and ethical issues that must be overcome. Additionally, animal bioassays are frequently not fast enough: by the time toxins are detected, fish populations are often already devastated.

The last decade has seen an increase in the number of molecular methods developed for monitoring algal abundance in natural waterways. One such method is quantitative real-time polymerase chain reaction (qRT-PCR), which comes with an unrivalled sensitivity and specificity. qRT-PCR has previously been used successfully to monitor blooms of *P. parvum* [59,60], and we have now begun to incorporate qRT-PCR as a regular monitoring application of *P. parvum* on Hickling Broad. Furthermore, sequence data from the PpDNAV-BW1 genome have provided us with the tools needed to monitor viral abundance alongside algal abundance (ongoing investigation), which will allow us to understand more about *P. parvum* bloom dynamics.

Current management methods for HABs range from the use of clay flocculants [61], algaecides [62], and even the manual relocation of affected fish to safer waterways. The local Environment Agency and volunteers were able to successfully save ∼230 000 fish through relocation during the *P. parvum* bloom on Hickling in 2015 [63]. However, this strategy is used as a last resort and is extremely time-consuming and labour-intensive. One alternative is the use of hydrogen peroxide as a chemical algaecide. Although hydrogen peroxide has not previously been used to tackle blooms of *P. parvum*, it has been used to effectively treat blooms of cyanobacteria [64] and toxin-producing dinoflagellates [65]. We are now working closely with local governing bodies to introduce hydrogen peroxide as a strategy in the management of *P. parvum* blooms (ongoing work).

## Concluding remarks

It is clear that *P. parvum* poses a major threat to ecosystems and the economies of the affected areas worldwide. These issues are only likely to increase in a warming climate. Despite this, there are still fundamental gaps in knowledge surrounding bloom dynamics of this alga. The discovery of a novel lytic virus that infects this organism has opened doors to answering questions about how viruses impact toxic HABs. Furthermore, the recent establishment of protocols for the detection of the ladder-frame polyether prymnesins means that *P. parvum* and PpDNAV-BW1 can now be used as a novel algae–virus system to answer a previously unanswerable question: how does viral infection affect toxin production and release in harmful algae?

As HABs increase in frequency, there is an even greater need for practical solutions. While carrying out this fundamental research, we have explored the use of qRT-PCR as a means for detecting *P. parvum* and its lytic virus, PpDNAV-BW1, which may ultimately be used as an early warning system for *P. parvum* blooms. Providing waterways lie close to arable land and are vulnerable to nutrient run-off, HABs are likely to occur. Therefore, we are now exploring the use of hydrogen peroxide as a cheap and effective management strategy.

## Abbreviations

HAB: harmful algal bloom
PpDNAV-BW1: *Prymnesium parvum* DNA virus
PSU: practical salinity units
SEM: scanning electron microscopy
TEM: transmission electron microscopy
